# *GLI1 *genotypes do not predict basal cell carcinoma risk: a case control study

**DOI:** 10.1186/1476-4598-8-113

**Published:** 2009-11-30

**Authors:** Andrea Watson, Paul Kent, Murad Alam, Amy S Paller, David M Umbach, Joon Won Yoon, Philip M Iannaccone, David O Walterhouse

**Affiliations:** 1 Department of Pediatrics, University of Minnesota - Duluth, Minnesota, USA; 2 Department of Pediatrics, Rush University Medical Center, Chicago, Illinois, USA; 3 Department of Dermatology, Northwestern University Feinberg School of Medicine, Chicago, Illinois, USA; 4 Biostatistics Branch, National Institute of Environmental Health Sciences, National Institutes of Health, Research Triangle Park, North Carolina, USA; 5 Department of Pediatrics, Northwestern University, Feinberg School of Medicine and the Developmental Biology Program of the Children's Memorial Research Center, Chicago Illinois, USA

## Abstract

**Background:**

Susceptibility to basal cell carcinoma results from complex interactions between ultraviolet radiation exposure and genetic factors. The *GLI1 *oncogene is believed to play a role in the genesis of these tumors. We determined whether *GLI1 *polymorphisms were risk factors for developing basal cell carcinoma, either alone or in combination with patterns of past sun exposure, and whether there were functional differences among different *GLI1 *haplotypes.

**Results:**

*GLI1 *genotypes at c.2798 and c.3298 from 201 basal cell carcinoma patients were compared to 201 age and sex-matched controls. Neither genotype nor haplotype frequencies differed between cases and controls. However, the odds of developing basal cell carcinoma on the trunk compared to the head/neck appeared somewhat lower with carriers of the c.3298GC than the CC genotype. There was no evidence for interactions between skin type, childhood sunburning, average adult sun exposure, adult sunbathing, or intermittency of sun exposure and *GLI1 *haplotype. Additionally, we found no significant differences in transcription activation or cell transforming ability among the four *GLI1 *haplotypes.

**Conclusion:**

These results suggest that different *GLI1 *genotypes alone or in combination with past sun exposure patterns as assessed in this study do not affect basal cell carcinoma risk.

## Background

Basal cell carcinoma (BCC) is the most common malignancy in Caucasians. Although mortality associated with BCC is low, BCC accounts for significant morbidity and places a large burden on the health care system. Susceptibility to BCC is believed to result from complex interactions between environmental ultraviolet (UV) radiation exposure and genetic factors [1]. Polymorphisms in genes encoding detoxifying enzymes (cytochrome p450 and glutathione S-transferase), the melanocortin 1 receptor, Agouti signaling protein, tyrosinase, and Patched 1 (PTCH1) have been associated with BCC risk [2-5]. However, polymorphic loci in genes that determine susceptibility for many patients who develop BCC and account for the phenotypic variability of BCC remain to be identified.

Dysregulation of the Sonic hedgehog signal transduction pathway plays an important role in the pathogenesis of BCC, presumably based on constitutive activation of GLI family transcription factors [6-8]. Indeed, transgenic mice expressing *GLI1 *in cutaneous keratinocytes develop BCCs, providing evidence that GLI1 plays a role in the genesis of BCC [9].

Polymorphic loci of unknown functional significance have been identified in *GLI1*, including c.2651A>C (D884A), c.2798G>A (G933D), c.3034G>T (G1012V), and c.3298G>C (E1100Q) [10,11]. None of these loci lies in a known functional domain of *GLI1*, however, nucleotides c.2798, c.3034, and c.3298 flank the carboxy terminal acidic transactivation domain (c.3060 - c.3273) [12]. Theoretically, amino acid differences in these residues could affect the conformation or function of this domain and be associated with disease based on altered transcriptional regulation by GLI1. Since *GLI1 *expression appears to play a fundamental role in the genesis of BCC, we determined whether any of the *GLI1 *genotypes represent risk factors for developing BCC alone or in combination with past sun exposure patterns and whether functional differences exist among the haplotypes. We find that different *GLI1 *genotypes alone or in combination with past sun exposure patterns as assessed in this study do not affect BCC risk.

## Methods

### Study design and subjects

A frequency-matched case control study was conducted from February to November 2007. Controls were matched to cases on sex and age (20-39 years, 40-59, 60-69, ≥ 70). In an attempt to even the distribution among age categories, we chose broader intervals where we expected fewer cases and narrower ones where we expected more.

In accord with the Declaration of Helsinki protocols, institutional IRB approval was granted from the Northwestern University Feinberg School of Medicine and Children's Memorial Hospital prior to study initiation. Written informed consent was obtained from each subject prior to enrollment. Eligible cases were recruited from non-Hispanic Caucasian patients presenting to the Northwestern Memorial Faculty Foundation Dermatology clinic for Mohs resection of histologically confirmed non-metastatic BCC. Eligible controls that had no history of skin cancer were also recruited from non-Hispanic Caucasian patients presenting to the same clinic. Individuals who had a known genetic disease or syndrome were excluded. Information collected included age, sex, skin type, and BCC location. Fitzpatrick skin type was ascertained by the patient's response to a standardized question [13]. BCC location was recorded as head/neck, trunk, or extremity.

### Single Nucleotide Polymorphism (SNP) analysis

Two buccal swabs (Whatman Omniswab, Florham Park, NJ) were obtained from consenting cases and controls. Genomic DNA was extracted using the QIAmp DNA mini kit (Qiagen, Valencia, CA). Two SNPs in the *GLI1 *gene (c.2798G>A and c.3298G>C) were analyzed via matrix-assisted laser desorption ionization time-of-flight mass spectrometry (MALDI-TOF MS) by the Translational Genomics Research Institute (TGen, Phoenix, AZ). The c.2651A>C (D884A) SNP was not analyzed based on its rare occurrence. The c.3034G>T (G1012V) SNP was not analyzed because it has only been observed in the Asian population [11].

### Assessment of sun exposure

A validated sun exposure questionnaire was completed by cases (N = 201) and controls (N = 201) in clinic [14-17]. Estimates for each of four aspects of sun exposure were determined (1. average adult sun exposure, 2. adult sunbathing score, 3. childhood sunburning, and 4. intermittency score). A measure of the lifetime average sun exposure in hours/day beginning at age 20 years is referred to as the average adult sun exposure. Within each age range a weighted average number of hours of exposure per day was calculated. These averages were combined across the age ranges as a weighted average with weights proportional to the number of years the subject spent in each age range. The adult sunbathing score serves as a measure of time that is spent with a large area of the body exposed to the sun. Nominal categories were converted to a numerical score for each age range as never = 0, rarely = 1, occasionally = 2, and often = 3. A weighted average of a subject's age-range-specific scores was calculated based on the number of years spent in each age range. Childhood sunburning was defined as erythema for ≥48 hours or blistering and recorded as yes or no. The intermittency score provides a measure of the proportion of sun exposure received on non-working days. Intermittency was assessed based on the absolute value of the difference between the weekend and weekday hours of exposure for a subject. An absolute value was determined for each of the age ranges in which a subject had spent time, and the values were combined across the age ranges as a weighted value based on the number of years the subject spent in each age range.

### Functional analysis

For CAT assays, *GLI1 *cDNA expression constructs that included nucleotides 231 through the polyA tail were prepared in the pM plasmid (Clontech, Palo Alto, CA) for each of four haplotypes (c.2798G>A and c.3298G>C). The sequence of all constructs was confirmed by automated sequence analysis. A total of 4 μg of DNA was transfected into HeLa cells in each experiment using Lipofectamine reagent (Gibco-BRL, Rockville, MD), including 1 μg of pG5CAT reporter plasmid (Clontech, Palo Alto, CA), 1 μg of pSV-β-galactosidase (Promega, Madison, WI), 0-250 ng of *GLI1 *effector plasmid, and pBluescript carrier DNA (Stratagene, La Jolla, CA) in an amount to make up the difference. CAT assays were performed as described by the manufacturer (Clontech, Palo Alto, CA). CAT activity was quantitated by scintillation counter and was normalized by measuring β-galactosidase activity spectrophotometrically.

For transformation assays, pLTR-*GLI1 *expression constructs containing each of the haplotypes in the pLTR-2 vector were prepared [18]. The sequence of all constructs was confirmed by automated sequence analysis. RK3E cells (American Type Culture Collection CRL1895, Manassas, VA) were transfected with 3 μg of each of the pLTR-*GLI1 *constructs using 12 μl of Lipofectamine reagent in 0.3 ml of OptiMEM (Gibco-BRL, Rockville, MD) [18]. Cells were incubated for 5 hr at 37°C before the OptiMEM was replaced with fresh culture media. Cells were incubated for two to four weeks and foci were counted.

### Statistical analysis

Hardy-Weinberg Equilibrium at each locus and estimates of linkage disequilibrium between loci were assessed using standard methods [19]. We assessed associations between BCC and potential risk factors (e.g., genotypes or sun exposure variables) using unconditional logistic regression models that adjusted for the matching variables. Interactions between potential risk factors were assessed similarly. We assessed associations between BCC location and genotype or exposure variables using unconditional polytomous logistic regression models applied only to cases. These models regarded the three possible locations as outcomes and the other variables as predictors. These polytomous models were also adjusted for the matching variables. Both the case-control logistic regression and case-only polytomous logistic regression measured associations with odds ratios (OR), and provided corresponding confidence intervals (CI) and two-tailed statistical tests. All regression calculations were carried out with SAS version 9.1 statistical software (SAS Institute, Inc., Cary, NC).

Analyses of possible associations of sun exposure variables or genotype with skin type were always adjusted for case-control status. Association of childhood sunburning with skin type was assessed with the Cochran-Armitage trend test. Associations of continuous sun exposure variables with skin type were assessed using tests for linear trend embedded in an analysis-of-variance model. Associations of *GLI1 *genotypes with skin type were assessed with the Cochran-Mantel-Haenszel test. These calculations were carried out with either SAS version 9.1 or with StatXact 6 (Cytel Software Corporation, Cambridge, MA).

## Results

### Characteristics of cases and controls

419 of 443 subjects (95%) interviewed met eligibility criteria, and 402 of 419 eligible individuals (96%) were enrolled. Buccal swabs, assessment of Fitzpatrick skin type, and sun-exposure questionnaires were obtained from 201 cases and 201 controls. Controls were frequency-matched to cases for age and sex (Table 1). The slight matching imbalances arose from an excess of female controls in the 40-59 age class and an excess of cases of both sexes in the ≥70 age class. The location of the BCC was head/neck in 157 (78%), trunk in 25 (12%), and extremity in 19 (10%). With the limited data available, we saw no evidence that the site distribution was related to either age or to sex.

**Table 1:** Matching of Cases and Controls for Age and Sex

	**Cases**		**Controls**	
	**N**	**%**	**N**	**%**
	
Total	201	100	201	100
				
Age (years)				
20-39	14	7	10	5
40-59	55	27	65	32
60-69	56	28	58	29
≥ 70	76	38	68	34
				
Sex				
female	91	45	96	48
male	110	55	105	52

### *GLI1* genotype and haplotype frequencies among cases and controls

*GLI1 *genotype distributions for nucleotides c.2798 and c.3298 for both the cases and controls were consistent with Hardy-Weinberg Equilibrium (cases: c.2798 p = 0.4, c.3298 p = 0.8; controls: c.2798 p = 0.06, c.3298 p = 0.2). The genotype frequencies were not significantly different between the cases and controls at c.2798 or c.3298 whether adjusted for age and sex or not (Additional file 1). Estimated haplotype frequencies were also not significantly different. Since we found no evidence for an association between haplotype and BCC, we combined cases and controls to estimate haplotype frequencies for the population; c.2798A;c.3298C = 0.58, c.2798A;c.3298G = 0.001, c.2798G;c.3298C = 0.08, and c.2798G;c.3298G = 0.34.

To estimate variability in *GLI1 *genotype distributions among different control groups, we compared the c.2798 and c.3298 genotype frequencies in our controls with a published historic control group consisting of a healthy Australian population with no evidence of BCC or other cancers [10]. We did not find evidence for any differences in the genotype distribution between our controls and the historic controls at c.2798 (p = 0.6). However, we found that the genotype distribution at c.3298 differed between the two groups (p = 0.006).

It has been suggested that different mechanisms mediate the development of truncal BCC compared with BCC on other sites. Therefore, we looked for associations between *GLI1 *genotype with primary site. We found no evidence for an association between the c.2798 genotype and BCC location (Additional file 2). However, the odds of developing BCC on the trunk compared with the head/neck region was lower for carriers of the GC than the CC genotype at c.3298. The power of this association is limited by the small number of truncal cases.

### Skin type and sun exposure estimates for cases and controls

Skin type distribution (N = 402) was not significantly different among cases and controls, whether adjusted for age and sex or not (Additional file 3). Although OR estimates point in the direction of a slight protective effect against developing BCC for individuals with skin types III + IV compared to I + II, we do not have evidence that skin type is associated with BCC (p = 0.32 when adjusted for age and sex). Skin type was not associated with BCC location (Additional file 4), and we did not find evidence for interactions between skin type and *GLI1 *genotype that affect BCC risk (Additional file 5).

There was not an association between any of the sun-exposure variables and BCC or between the adult sunbathing score or childhood sunburning and BCC location whether adjusted for age and sex or not (Additional files 3, 4). Longer average adult sun exposure appeared to reduce the odds of BCC developing on the trunk compared to the head/neck (p = 0.05) (Additional file 4), while intermittency appeared to be associated with BCC site (p = 0.04) and with increased odds of developing BCC on the extremities compared to the head/neck (p = 0.02) (Additional file 4). There was not evidence for interactions between patterns of past sun exposure and either *GLI1 *genotype or haplotype (data not shown).

### Functional analyses of the different *GLI1* haplotypes

**Figure 1:**
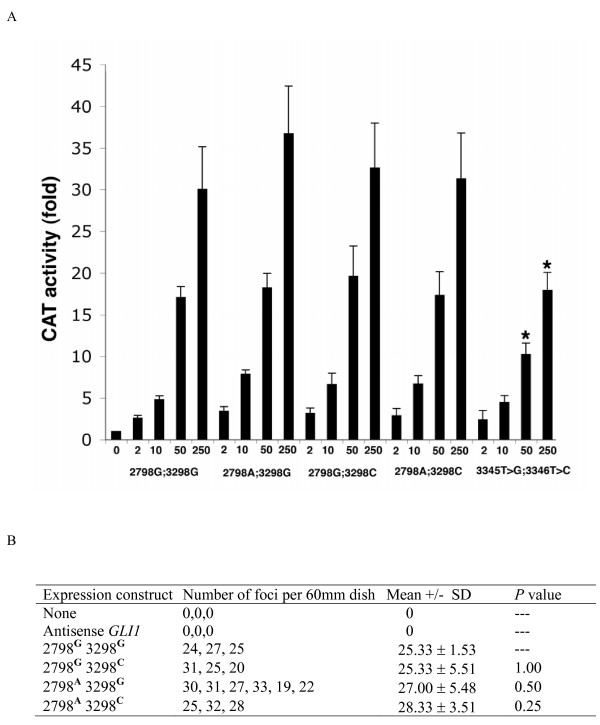
**Different *GLI1 *haplotypes function similarly**. **A. Different *GLI1 *haplotypes activate transcription of a CAT reporter comparably in HeLa cells**. The *GLI1 *haplotypes are indicated along the abscissa with the amount transfected (ng) in each experiment. CAT activity, normalized by measuring β-galactosidase activity spectrophotometrically (Promega, Madison, WI), is indicated on the ordinate. Bars represent the means derived from three independent experiments. *indicates p < 0.05 calculated using a test of difference between means, comparing 2798G;3298G with the corresponding amount of each of the other haplotypes. **B. Different *GLI1 *haplotypes transform RK3E cells comparably**. The p value represents a test of difference between means, comparing 2798G;3298G with each of the other *GLI1 *haplotypes.

We tested the ability of each of the *GLI1 *haplotypes to activate transcription and transform RK3E cells in culture. We found no significant differences in activity among the four haplotypes by either of these assays (Figure 1). Introduction of point mutations that change a specific residue in the acidic transactivation domain of GLI1 significantly reduced GLI1-induced transactivation of the pG5CAT reporter (c.3345 T>G and c.3346 T>C [F1048A]), demonstrating that the assay is sensitive enough to detect functional differences caused by single amino acid changes. These results support the conclusion that different *GLI1 *haplotypes function similarly.

## Discussion

We did not find evidence that different *GLI1 *genotypes alone or in combination with past sun exposure patterns affect BCC risk. Additionally, we found no significant differences in transcription activation or cell transforming ability among the four *GLI1 *haplotypes.

The genotype distribution of our controls differed from a historic control group at c.3298. It is unclear whether the differences represent variation within the US Midwest population or inherent differences in the genetic distribution between distinct populations world-wide. In either case, it will be important to carefully select internal controls for future studies that assess *GLI1 *genotype frequencies.

It is not entirely surprising that skin type distribution and sun exposure estimates did not differ between our cases and controls since associations between these and BCC have been inconsistently reported [14,16,17]. We recognize that recall bias may limit the validity of the sun-exposure estimates and reduce their value in determining associations between BCC and specific past sun exposure patterns. Similar to other studies, participants self-administered the questionnaire in clinic to reduce the introduction of bias. In contrast to the other studies, we compared BCC patients to individuals who were generally well and without BCC. It is not clear why this comparison would give negative results. It is also possible that perceptions of sunbathing practices or genetic heterogeneity differ between individuals in different populations, sometimes limiting the discriminating value of the questionnaire. Finally, it is possible that UV radiation played a limited role in the development of BCC in the population that was assessed.

## Conclusion

*GLI1 *genotypes function similarly and do not affect BCC risk either alone or in combination with past sun exposure patterns.

## Competing interests

The authors declare that they have no competing interests.

## Authors' contributions

AW collected patient data, participated in data analysis, and drafted the manuscript. PK collected patient data. MA participated in patient data collection, study design, and manuscript revision. AP participated in study design, and manuscript revision. DU contributed to study design, completed data analysis, participated in data interpretation, and helped to draft and revise the manuscript. JY carried out the transcription and transformation assays, and participated in manuscript revision. PI participated in study design, data analysis, data interpretation, and manuscript revision. DW conceived of the study, participated in study design, data analysis, data interpretation, and manuscript revision. All authors read and approved the final manuscript.

## Authors' Information

AW, PK, and DW are pediatric oncologists. AP is a dermatologist. MA is a dermatologist and cutaneous surgeon. JY is a developmental biologist. PI is a pathologist and developmental biologist. DU is a statistician.

## Supplementary Material

Additional file 1 Association of *GLI1 *genotypes with BCC. Click here for file

Additional file 2 Association of *GLI1 *genotypes with BCC site. Click here for file

Additional file 3 Association of sun-exposure variables with BCC. Click here for file

Additional file 4 Association of sun exposure variables with BCC site (adjusted for age and sex). Click here for file

Additional file 5 Association of BCC with cross-classification of *GLI1 *genotypes and dichotomized skin type. Click here for file
