# Supplementary material for: GLI1 genotypes do not predict basal cell carcinoma risk: a case control study
Source: Mol Cancer. 2009 Nov 30;8:113. doi: 10.1186/1476-4598-8-113 (PMC2789726; doi:10.1186/1476-4598-8-113)
Supplement: Additional file 1 — Association of GLI1 genotypes with BCC. [file 1476-4598-8-113-S1.DOC]

**Additional file 1** Association of *GLI1* genotypes with BCC

| Variable | Cases |  | Controls |  | Crude | | | Adjusted for age and sex | | |
| --- | --- | --- | --- | --- | --- | --- | --- | --- | --- | --- |
|  | N | % | N | % | OR | 95% CI | p-value | OR | 95% CI | p-value |
|  |  |  |  |  |  |  |  |  |  |  |
| Total | 201 | 100% | 201 | 100% |  |  |  |  |  |  |
|  |  |  |  |  |  |  |  |  |  |  |
| c.2798 |  |  |  |  |  |  |  |  |  |  |
| AA | 66 | 32% | 59 | 29% | ref |  |  | ref |  |  |
| GA | 102 | 51% | 106 | 53% | 0.86 | 0.55, 1.34 | 0.51 | 0.86 | 0.55, 1.34 | 0.50 |
| GG | 31 | 16% | 27 | 13% | 1.03 | 0.55, 1.92 | 0.93 | 1.02 | 0.54, 1.91 | 0.95 |
| na^1^ | 2 | 1% | 9 | 5% | -- |  |  | -- |  |  |
|  |  |  |  |  |  | 2 df test | 0.73 |  |  | 0.74 |
|  |  |  |  |  |  | trend test | 0.89 |  |  | 0.87 |
| c.3298 |  |  |  |  |  |  |  |  |  |  |
| CC | 84 | 42% | 85 | 42% | ref |  |  | ref |  |  |
| GC | 92 | 46% | 94 | 47% | 0.99 | 0.65, 1.50 | 0.96 | 0.99 | 0.65, 1.50 | 0.96 |
| GG | 23 | 11% | 17 | 8% | 1.37 | 0.68, 2.75 | 0.38 | 1.33 | 0.66, 2.68 | 0.42 |
| na^1^ | 2 | 1% | 5 | 3% | -- |  |  | -- |  |  |
|  |  |  |  |  |  | 2 df test | 0.64 |  |  | 0.69 |
|  |  |  |  |  |  | trend test | 0.54 |  |  | 0.58 |

^1^genotyping failed in these individuals
