# Supplementary material for: GLI1 genotypes do not predict basal cell carcinoma risk: a case control study
Source: Mol Cancer. 2009 Nov 30;8:113. doi: 10.1186/1476-4598-8-113 (PMC2789726; doi:10.1186/1476-4598-8-113)
Supplement: Additional file 2 — Association of GLI1 genotypes with BCC site. [file 1476-4598-8-113-S2.DOC]

**Additional file 2** Association of *GLI1* genotypes with BCC site

| Variable | Total Cases | | Head/Neck | | Extremities | | Trunk | | Extremities vs. Head/Neck | | | Trunk vs. Head/Neck | | |
| --- | --- | --- | --- | --- | --- | --- | --- | --- | --- | --- | --- | --- | --- | --- |
|  | N | % | N | % | N | % | N | % | OR | 95% CI | p-value | OR | 95% CI | p-value |
|  |  |  |  |  |  |  |  |  |  |  |  |  |  |  |
| c.2798 |  |  |  |  |  |  |  |  |  |  |  |  |  |  |
| AA | 66 | 33% | 49 | 31% | 5 | 28% | 12 | 48% | ref |  |  | ref |  |  |
| GA | 102 | 51% | 82 | 53% | 10 | 55% | 10 | 40% | 1.20 | 0.39, 3.70 | 0.76 | 0.50 | 0.20, 1.24 | 0.13 |
| GG | 31 | 16% | 25 | 16% | 3 | 17% | 3 | 12% | 1.18 | 0.26, 5.33 | 0.83 | 0.49 | 0.13, 1.90 | 0.30 |
| total | 199 | 100% | 156 | 100% | 18 | 100% | 25 | 100% |  |  |  |  |  |  |
|  |  |  |  |  | 4-df test | | p=0.58 | |  | 2 df test | 0.95 |  |  | 0.27 |
|  |  |  |  |  |  |  |  |  | 1-df trend test | | 0.80 |  |  | 0.16 |
| c.3298 |  |  |  |  |  |  |  |  |  |  |  |  |  |  |
| CC | 84 | 42% | 62 | 40% | 6 | 32% | 16 | 64% | ref |  |  | ref |  |  |
| GC | 92 | 46% | 74 | 48% | 12 | 63% | 6 | 24% | 1.68 | 0.59, 4.72 | 0.33 | 0.31 | 0.12, 0.85 | 0.02 |
| GG | 23 | 11% | 19 | 12% | 1 | 5% | 3 | 12% | 0.54 | 0.06, 4.80 | 0.58 | 0.61 | 0.16, 2.33 | 0.47 |
| total | 199 | 100% | 155 | 100% | 19 | 100% | 25 | 100% |  |  |  |  |  |  |
|  |  |  |  |  | 4-df test | | p=0.11 | |  | 2 df test | 0.42 |  |  | 0.07 |
|  |  |  |  |  |  |  |  |  | 1-df trend test | | 0.93 |  |  | 0.10 |

2 subjects missing genotyping data were excluded for each locus
