# Supplementary material for: GLI1 genotypes do not predict basal cell carcinoma risk: a case control study
Source: Mol Cancer. 2009 Nov 30;8:113. doi: 10.1186/1476-4598-8-113 (PMC2789726; doi:10.1186/1476-4598-8-113)
Supplement: Additional file 3 — Association of sun-exposure variables with BCC. [file 1476-4598-8-113-S3.DOC]

**Additional file 3** Association of sun-exposure variables with BCC

| Variable | Cases |  | Controls |  | Crude | | | Adjusted for age and sex | | |
| --- | --- | --- | --- | --- | --- | --- | --- | --- | --- | --- |
|  | N | % | N | % | OR | 95% CI | p-value | OR | 95% CI | p-value |
|  |  |  |  |  |  |  |  |  |  |  |
| Total | 201 | 100% | 201 | 100% |  |  |  |  |  |  |
|  |  |  |  |  |  |  |  |  |  |  |
| Skin type |  |  |  |  |  |  |  |  |  |  |
| I | 29 | 14.5% | 36 | 18% | ref |  |  | ref |  |  |
| II | 70 | 35% | 55 | 27% | 1.58 | 0.86, 2.89 | 0.14 | 1.58 | 0.86, 2.90 | 0.14 |
| III | 75 | 37% | 70 | 35% | 1.33 | 0.74, 2.39 | 0.34 | 1.28 | 0.71, 2.33 | 0.41 |
| IV | 27 | 13.5% | 40 | 20% | 0.84 | 0.42, 1.67 | 0.62 | 0.76 | 0.37, 1.54 | 0.44 |
|  |  |  |  |  |  | 3-df test: | 0.16 |  |  | 0.10 |
|  |  |  |  |  |  | trend test: | 0.46 |  |  | 0.33 |
| I + II | 99 | 49.3% | 91 | 45.3% | ref |  |  | ref |  |  |
| III + IV | 102 | 50.7% | 110 | 54.7% | 0.85 | 057, 1.26 | 0.42 | 0.81 | 0.54, 1.22 | 0.32 |
|  |  |  |  |  |  |  |  |  |  |  |
| Childhood sunburning |  |  |  |  |  |  |  |  |  |  |
| yes | 138 | 69% | 134 | 67% | 1.10 | 0.72, 1.67 | 0.67 | 1.14 | 0.75, 1.75 | 0.54 |
| no | 62 | 31% | 66 | 33% | ref |  |  | ref |  |  |
| na | 1 | ~ | 1 | ~ |  |  |  |  |  |  |
|  |  |  |  |  |  |  |  |  |  |  |
|  |  |  |  |  |  |  |  |  |  |  |
|  | Mean (median) | Standard deviation | Mean  (median) | Standard Deviation |  |  |  |  |  |  |
| Average adult sun exposure (hr/day) | 2.16  (1.79) | 1.57 | 2.08  (1.66) | 1.67 | 1.03 | 0.92, 1.17 | 0.59 | 1.03 | 0.91, 1.17 | 0.63 |
|  |  |  |  |  |  |  |  |  |  |  |
| Adult sunbathing score | 1.44  (1.57) | 0.90 | 1.46  (1.45) | 0.89 | 0.98 | 0.79, 1.22 | 0.84 | 1.00 | 0.80, 1.25 | 0.97 |
|  |  |  |  |  |  |  |  |  |  |  |
| Intermittency score | 1.72  (1.50) | 1.39 | 1.89  (1.40) | 1.76 | 0.93 | 0.83, 1.06 | 0.28 | 0.94 | 0.82, 1.07 | 0.32 |
