# Supplementary material for: GLI1 genotypes do not predict basal cell carcinoma risk: a case control study
Source: Mol Cancer. 2009 Nov 30;8:113. doi: 10.1186/1476-4598-8-113 (PMC2789726; doi:10.1186/1476-4598-8-113)
Supplement: Additional file 4 — Association of sun exposure variables with BCC site (adjusted for age and sex). [file 1476-4598-8-113-S4.DOC]

**Additional file 4** Association of sun exposure variables with BCC site (adjusted for age and sex)

| Variable | Total Cases | | Head/Neck | | Extremities | | | Trunk | | | Extremities vs. Head/Neck | | | Trunk vs. Head/Neck | | |
| --- | --- | --- | --- | --- | --- | --- | --- | --- | --- | --- | --- | --- | --- | --- | --- | --- |
|  | N | % | N | % | N | % | | N | % | | OR | 95% CI | p-value | OR | 95% CI | p-value |
|  |  |  |  |  |  |  | |  |  | |  |  |  |  |  |  |
| Childhood sunburning | |  |  |  |  |  | |  |  | |  |  |  |  |  |  |
| yes | 138 | 69% | 108 | 69% | 12 | 63% | | 18 | 72% | | 0.75 | 0.27, 2.10 | 0.58 | 0.92 | 0.35, 2.44 | 0.87 |
| no | 62 | 31% | 48 | 31% | 7 | 37% | | 7 | 18% | | ref |  |  | ref |  |  |
| total | 200 |  | 156 |  | 19 |  | | 25 |  | |  |  |  |  |  |  |
|  |  |  |  |  | 2-df test | | | p=0.82 | | |  |  |  |  |  |  |
|  |  |  |  |  |  |  | |  |  | |  |  |  |  |  |  |
| Skin type |  |  |  |  |  |  | |  |  | |  |  |  |  |  |  |
| I | 29 | 15% | 21 | 13% | 4 | 21% | | 4 | 16% | | ref |  |  | ref |  |  |
| II | 70 | 35% | 54 | 34% | 6 | 32% | | 10 | 40% | | 0.66 | 0.17, 2.60 | 0.55 | 1.06 | 0.29, 3.88 | 0.93 |
| III | 75 | 37% | 59 | 38% | 8 | 42% | | 8 | 32% | | 0.78 | 0.21, 2.96 | 0.71 | 0.82 | 0.22, 3.11 | 0.77 |
| IV | 27 | 13% | 23 | 15% | 1 | 5% | | 3 | 12% | | 0.24 | 0.03, 2.42 | 0.23 | 1.03 | 0.19, 5.51 | 0.97 |
| total | 201 |  | 157 |  | 19 |  | | 25 |  | |  |  |  |  |  |  |
|  |  |  |  |  | 6-df test | | | p=0.93 | | |  | 3-df test | 0.67 |  |  | 0.97 |
|  |  |  |  |  |  | |  |  | |  |  |  |  |  |  |  |
|  | Mean (med^1^) | Std dev | Mean (med^1^) | Std dev | Mean (med^1^) | | Std dev | Mean (med^1^) | | Std dev |  |  |  |  |  |  |
| Average adult sun exposure (hr/day) | 2.16  (1.79) | 1.57 | 2.26  (1.81) | 1.63 | 2.25  (1.86) | | 1.41 | 1.55  (1.29) | | 1.14 | 0.98 | 0.72, 1.34 | 0.91 | 0.67 | 0.45, 0.99 | 0.05 |
|  |  |  |  |  | 2-df test | | | p=0.14 | | |  |  |  |  |  |  |
|  |  |  |  |  |  |  | |  |  | |  |  |  |  |  |  |
| Adult sunbathing score | 1.44  (1.57) | 0.90 | 1.38  (1.45) | 0.93 | 1.58  (1.57) | 0.83 | | 1.71 (1.90) | 0.70 | | 1.24 | 0.71, 2.18 | 0.45 | 1.32 | 0.78, 2.24 | 0.30 |
|  |  |  |  |  | 2-df test | | | p=0.48 | | |  |  |  |  |  |  |
|  |  |  |  |  |  |  | |  |  | |  |  |  |  |  |  |
| Intermittency score | 1.72  (1.50) | 1.39 | 1.63  (1.38) | 1.42 | 2.65  (2.75) | 1.30 | | 1.61  (1.30) | 0.97 | | 1.49 | 1.07, 2.08 | 0.02 | 0.94 | 0.65, 1.36 | 0.75 |
|  |  |  |  |  | 2-df test | | | p=0.04 | | |  |  |  |  |  |  |
|  |  |  |  |  |  |  | |  |  | |  |  |  |  |  |  |

^1^med = median
