# Supplementary material for: GLI1 genotypes do not predict basal cell carcinoma risk: a case control study
Source: Mol Cancer. 2009 Nov 30;8:113. doi: 10.1186/1476-4598-8-113 (PMC2789726; doi:10.1186/1476-4598-8-113)
Supplement: Additional file 5 — Association of BCC with cross-classification of GLI1 genotypes and dichotomized skin type. [file 1476-4598-8-113-S5.DOC]

**Additional file 5** Association of BCC with cross-classification of *GLI1* genotypes and dichotomized skin type

| Variable | Cases |  | Controls |  | Crude | | | Adjusted for age and sex | | |
| --- | --- | --- | --- | --- | --- | --- | --- | --- | --- | --- |
|  | N | % | N | % | OR | 95% CI | p-value | OR | 95% CI | p-value |
|  |  |  |  |  |  |  |  |  |  |  |
| c.2798 |  |  |  |  |  |  |  |  |  |  |
| Skin type I+II AA | 34 | 34% | 23 | 25% | ref |  |  |  |  |  |
| GA | 47 | 47% | 56 | 62% | 0.57 | 0.29, 1.09 | 0.09 | 0.58 | 0.30, 1.11 | 0.10 |
| GG | 17 | 17% | 9 | 10% | 1.22 | 0.49, 3.36 | 0.62 | 1.26 | 0.48, 3.34 | 0.64 |
| na^1^ | 1 | 1% | 3 | 3% | -- | -- | -- | -- | -- | -- |
| total | 99 |  | 91 | 100% |  |  |  |  |  |  |
| Skin type III+IV AA | 32 | 31% | 36 | 33% | 0.60 | 0.30, 1.23 | 0.16 | 0.57 | 0.28, 1.20 | 0.14 |
| GA | 55 | 54% | 50 | 45.5% | 0.74 | 0.40, 1.43 | 0.38 | 0.71 | 0.37, 1.38 | 0.31 |
| GG | 14 | 14% | 18 | 16% | 0.53 | 0.22, 1.26 | 0.15 | 0.50 | 0.21, 1.23 | 0.13 |
| na^1^ | 1 | 1% | 6 | 5.5% | -- | -- | -- | -- | -- | -- |
| total | 102 |  | 110 | 100% |  |  |  |  |  |  |
|  |  |  |  |  | 5-df overall test | | 0.29 |  |  | 0.30 |
|  |  |  |  |  | 2-df interaction test | | 0.08 |  |  | 0.09 |
|  |  |  |  |  |  |  |  |  |  |  |
| c.3298 |  |  |  |  |  |  |  |  |  |  |
| Skin type I+II CC | 41 | 42% | 37 | 41% | ref |  |  |  |  |  |
| GC | 45 | 46% | 47 | 52% | 0.86 | 0.47, 1.58 | 0.64 | 0.88 | 0.48, 1.61 | 0.67 |
| GG | 13 | 11% | 5 | 5% | 2.34 | 0.76, 7.21 | 0.14 | 2.32 | 0.75, 7.16 | 0.14 |
| na^1^ | 0 | 1% | 2 | 2% | -- | -- | -- | -- | -- | -- |
| total | 99 |  | 91 |  |  |  |  |  |  |  |
| Skin type III+IV CC | 43 | 42% | 48 | 44% | 0.81 | 0.44, 1.48 | 0.49 | 0.79 | 0.43, 1.46 | 0.45 |
| GC | 47 | 46% | 47 | 43% | 0.90 | 0.49, 1.65 | 0.74 | 0.87 | 0.47, 1.59 | 0.64 |
| GG | 10 | 10% | 12 | 11% | 0.75 | 0.29, 1.94 | 0.56 | 0.70 | 0.27, 1,84 | 0.47 |
| na^1^ | 2 | 2% | 3 | 3% | -- | -- | -- | -- | -- | -- |
| total | 102 |  | 110 |  |  |  |  |  |  |  |
|  |  |  |  |  | 5-df overall test | | 0.55 |  |  | 0.53 |
|  |  |  |  |  | 2-df interaction test | | 0.27 |  |  | 0.28 |

^1^ genotyping failed in these individuals
